# Development and validation of a new tool to estimate early mortality in patients with advanced cancer treated with immunotherapy

**DOI:** 10.1007/s00262-024-03836-w

**Published:** 2024-10-03

**Authors:** Andrea De Giglio, Alessandro Leonetti, Francesca Comito, Daria Maria Filippini, Veronica Mollica, Karim Rihawi, Marianna Peroni, Giulia Mazzaschi, Ilaria Ricciotti, Francesca Carosi, Andrea Marchetti, Matteo Rosellini, Ambrogio Gagliano, Valentina Favorito, Elisabetta Nobili, Francesco Gelsomino, Barbara Melotti, Paola Valeria Marchese, Francesca Sperandi, Alessandro Di Federico, Sebastiano Buti, Fabiana Perrone, Francesco Massari, Maria Abbondanza Pantaleo, Marcello Tiseo, Andrea Ardizzoni

**Affiliations:** 1 Department of Medical and Surgical Sciences, Alma Mater Studiorum University of Bologna, Via Massarenti, 9, 40138 Bologna, Italy; 2 Medical Oncology, IRCCS Azienda Ospedaliero-Universitaria Di Bologna, Bologna, Italy; 3 Medical Oncology Unit, University Hospital of Parma, Parma, Italy; 4 Department of Medicine and Surgery, University of Parma, Parma, Italy

**Keywords:** Immunotherapy, Early mortality, Solid tumors, Prognostic prediction

## Abstract

**Background:**

Immune checkpoint inhibitors (ICIs) are standard treatments for advanced solid cancers. Resistance to ICIs, both primary and secondary, poses challenges, with early mortality (EM) within 30–90 days indicating a lack of benefit. Prognostic factors for EM, including the lung immune prognostic index (LIPI), remain underexplored.

**Methods:**

We performed a retrospective, observational study including patients affected by advanced solid tumors, treated with ICI as single agent or combined with other agents. Logistic regression models identified factors associated with EM and 90-day progression risks. A nomogram for predicting 90-day mortality was built and validated within an external cohort.

**Results:**

In total, 637 patients received ICIs (single agent or in combination with other drugs) for advanced solid tumors. Most patients were male (61.9%), with NSCLC as the prevalent tumor (61.8%). Within the cohort, 21.3% died within 90 days, 8.4% died within 30 days, and 34.5% experienced early progression. Factors independently associated with 90-day mortality included ECOG PS 2 and a high/intermediate LIPI score. For 30-day mortality, lung metastasis and a high/intermediate LIPI score were independent risk factors. Regarding early progression, high/intermediate LIPI score was independently associated. A predictive nomogram for 90-day mortality combining LIPI and ECOG PS achieved an AUC of 0.76 (95% CI 0.71–0.81). The discrimination ability of the nomogram was confirmed in the external validation cohort (n = 255) (AUC 0.72, 95% CI 0.64–0.80).

**Conclusion:**

LIPI and ECOG PS independently were able to estimate 90-day mortality, with LIPI also demonstrating prognostic validity for 30-day mortality and early progression.

**Supplementary Information:**

The online version contains supplementary material available at 10.1007/s00262-024-03836-w.

## Introduction

Immune checkpoint inhibitors (ICIs) are currently the standard of care for many advanced solid cancers, either as a single agent or in combination with chemotherapy or molecular-targeted agents.

A considerable proportion of patients exhibit primary or secondary resistance to ICIs. Primary resistance is characterized by the lack of clinical or radiological benefit following at least six weeks of treatment [1]. Secondary resistance, on the other hand, is defined as clinical or radiological progression in a patient who had previously demonstrated a response to treatment or remained stable for longer than six months [1].

Different definitions have been provided to include the speed of progression, mainly derived from retrospective experiences. In this context, fast progression (FP) refers to a condition with an increase of at least 50% in the sum of the longest diameter of target lesions within six weeks from the starting point [2]. The concept of hyperprogressive (HPD) disease, which entails the dynamic evaluation of tumor growth, remains controversial owing to the lack of a unanimous consensus on its definition and prevalence [2, 3].

Despite the plethora of definitions regarding the patterns of progressive disease (PD), early mortality (EM) stands for death due to disease progression within 30–90 days from the treatment initiation [3].

According to findings from a large cohort study, patients with solid cancer treated with ICIs were observed to have a mortality rate of 7% within 30 days from treatment start, 15% within 60 days, and 22% within 90 days [3]. Evidently, these patients do not derive any benefit from immunotherapy and, if identified upfront, should ideally be spared this form of treatment since, in this case, immunotherapy would be associated only with useless costs and toxicity and, in addition, a possible detrimental effect on survival cannot be excluded.

Several potential prognostic factors have been investigated as predictors of ICI-related EM in different cancers, including age, primary tumor site (lung, head and neck), baseline laboratory values (hemoglobin, white blood cells, platelet count, neutrophil-to-lymphocyte ratio [NLR], lactate dehydrogenase [LDH], albumin, and Eastern Cooperative Oncology Group performance status [ECOG PS]) [4–6].

The lung immune prognostic index (LIPI), a score incorporating the derived NLR (dNLR) and serum LDH levels, demonstrated its prognostic value first in non-small cell lung cancer (NSCLC) [7]. Subsequent studies showed its association with disease progression and mortality risk in other tumor types, such as genitourinary [8, 9], breast [10], melanoma [9], and head and neck cancers [11], suggesting its agnostic applicability. No studies explored the short-term prognostic validity of the LIPI score.

The present study investigated clinical and laboratory factors, including LIPI score, associated with EM and early progression under ICI-based treatments. Moreover, we developed a nomogram to predict 90-day mortality with an external validation.

## Methods

We performed a single-center, retrospective, observational study including patients affected by advanced solid tumors (stage IV), treated with ICI as single agent or combined with other agents (chemotherapy, ICI [ICI doublets], targeted therapy) between August 2015 and December 2023 at the IRCCS Azienda Ospedaliero-Universitaria of Bologna, Italy. We collected data for a validation cohort of patients with the same clinical characteristics and treated within the same window of time at the Azienda Ospedaliero-Universitaria of Parma, Italy.

This study was conducted in accordance with the Declaration of Helsinki (1964) after obtaining approval from the local Ethics Committee. Data were manually collected from electronic and paper-based medical records. The variables recorded included age, sex, tumor histology, immunotherapy-based treatments (type, line, date of start and stop), ECOG PS, radiological findings at the beginning and throughout the study, number of metastatic sites, site of metastasis (other than primary tumor lesion), laboratory test (complete blood count, LDH), most recent follow-up, date of death. The LIPI score was collected at baseline (within the same blood test, 30 days from treatment start) for each patient, when available. This score considers two factors: the dNLR [neutrophils / (leukocytes minus neutrophils)] and serum LDH levels. A dNLR value greater than 3 or LDH levels above the upper limit of normal count for 1 point each. Based on the values of these two variables, patients are categorized into three prognostic groups: low risk (0 points), intermediate risk (1 point), and high risk (2 points).

The primary objective of this study was to identify baseline clinical and laboratory prognostic factors, including the LIPI score, associated with early 90-day mortality to ICI-based treatments.

A nomogram was developed by integrating two independent prognostic factors derived from the multivariate analysis for 90-day mortality prediction within the development cohort, and a score was computed based on the risk of EM associated with each variable included in the model. The validation cohort was used to test the nomogram’s performance.

The secondary objectives were to investigate prognostic factors of 30-day mortality and early disease progression (≤ 90 days from treatment initiation). The decision to use a 90-day cutoff for the primary and secondary objectives was based on literature data and clinical practice, where the initial radiological assessment is generally conducted after 3–4 cycles of treatment (12 weeks).

### Statistical methods

Clinical and laboratory findings were analyzed as continuous or categorical variables, with median values and proportions reported, as appropriate. The normality of the distribution was verified using the Shapiro test. To compare means and proportions, T-test (Pearson correlation test if needed) and Chi-squared test (or Fisher's exact test, if needed) were performed. Overall survival (OS) was defined as the time between the start of treatment and death from any cause. Progression-free survival (OS) was defined as the time from treatment initiation to the first clinical or radiographical evidence of disease progression or death from any cause. The ROC curve analysis was performed to determine the area under the curve (AUC) for the score obtained by nomogram analysis to differentiate between patients who survived and those who died within 90 days. A multivariable logistic regression model was employed to investigate the factors associated with EM, and subsequently, the adjusted odds ratios (ORs) and 95% confidence intervals (CIs) were reported. Patients alive with a follow-up inferior to 30 or 90 days were excluded from the logistic regression analyses. A statistically significant p-value was considered when < 0.05. Statistical analyses were accomplished with R-Studio free software, version 2023.06.2.

## Results

### Baseline characteristics and survival outcomes

A total of 637 patients were included in the training cohort. Most patients were males (61.9%) and had an ECOG PS of 0–1 (86.5%). NSCLC was the most frequent tumor type (61.8%), followed by melanoma (17.3%), head–neck (11.3%), genitourinary (6.4%), and gastrointestinal (3.1%) tumors. 68.4% of patients received ICI as single agent and were treated in first line (67%). Baseline characteristics are summarized in Table 1. The median OS in the training cohort was 11.7 months (95% CI 9.6–15.0), and the median follow-up time was 26.1 months (IQR 11.7–41.4). The median PFS in the training cohort was 5.6 months (95% CI 4.8–6.5). The Cox regression analyses for death and progression risk are summarized in Tables S1 and S2.

**Table 1:** Baseline characteristics of the training cohort

	Overall (N = 636)
Age	
≤ 65	238 (37.4%)
> 65	398 (62.6%)
Sex	
Female	242 (38.1%)
Male	394 (61.9%)
ECOG PS	
0–1	511 (80.3%)
2	78 (12.3%)
Missing	47 (7.4%)
Smoking status	
Current smoker	112 (17.6%)
Former smoker	274 (43.1%)
Never smoker	69 (10.8%)
Missing	181 (28.5%)
Histology	
Gastrointestinal	20 (3.1%)
Genitourinary	41 (6.4%)
Head–neck	72 (11.3%)
Melanoma	110 (17.3%)
NSCLC	393 (61.8%)
Num. of metastatic sites	
≤ 3	391 (61.5%)
> 3	76 (11.9%)
Missing	169 (26.6%)
Lung met	
No	291 (45.8%)
Yes	339 (53.3%)
Missing	6 (0.9%)
Brain met	
No	526 (82.7%)
Yes	105 (16.5%)
Missing	5 (0.8%)
Liver met	
No	514 (80.8%)
Yes	116 (18.2%)
Missing	6 (0.9%)
Line of treatment	
First	426 (67.0%)
Subsequent	210 (33.0%)
Type of treatment	
CT-ICI	171 (26.9%)
ICI-ICI	13 (2.0%)
ICI	435 (68.4%)
immuno-TKI	17 (2.7%)
dNLR	
Mean (SD)	2.99 (2.28)
Median [Min, Max]	2.36 [0.0318, 20.4]
Missing	35 (5.5%)
LIPI	
High	71 (11.2%)
Intermediate	188 (29.6%)
Low	230 (36.2%)
Missing	147 (23.1%)

*ECOG PS* Eastern Cooperative Oncology Group performance status, *NSCLC* non-small cell lung cancer; *Num*. number, *met*. Metastasis, *ICI* immune checkpoint inhibitor, *CT* chemotherapy, *TKI* tyrosine kinase inhibitor, *dNLR* derived neutrophil-to-lymphocyte ratio, *LIPI* lung immune prognostic index

### Early mortality and progression risk

One hundred and thirty-six patients (21.3%) died within 90 days. The distribution of baseline features according to 90-day mortality is reported in Table 2.

**Table 2:** Baseline characteristics according to 90-day mortality in the training cohort

	90-day mortality No (%)	90-day mortality Yes (%)	Total (%)	*p* value
Age				
> 65	293 (61.9)	92 (67.6)	385 (63.2)	0.265
≤ 65	180 (38.1)	44 (32.4)	224 (36.8)	
Sex				
Female	185 (39.1)	48 (35.6)	233 (38.3)	0.516
Male	288 (60.9)	87 (64.4)	375 (61.7)	
ECOG PS				
0–1	398 (91.1)	88 (70.4)	486 (86.5)	< 0.001
2	39 (8.9)	37 (29.6)	76 (13.5)	
Smoking history				
Current smoker	82 (25.4)	26 (22.8)	108 (24.7)	0.854
Former smoker	193 (59.8)	71 (62.3)	264 (60.4)	
Never smoker	48 (14.9)	17 (14.9)	65 (14.9)	
Histology				
Gastrointestinal	18 (3.8)	1 (0.7)	19 (3.1)	0.001
Genitourinary	29 (6.1)	4 (2.9)	33 (5.4)	
Head–neck	51 (10.8)	12 (8.8)	63 (10.3)	
Melanoma	97 (20.5)	13 (9.6)	110 (18.1)	
NSCLC	278 (58.8)	106 (77.9)	384 (63.1)	
Num. of metastatic sites				
> 3	51 (14.1)	22 (27.8)	73 (16.6)	0.005
≤ 3	311 (85.9)	57 (72.2)	368 (83.4)	
Lung met				
No	225 (47.9)	46 (34.6)	271 (44.9)	0.009
Yes	245 (52.1)	87 (65.4)	332 (55.1)	
Brain met				
No	403 (85.6)	101 (75.9)	504 (83.4)	0.012
Yes	68 (14.4)	32 (24.1)	100 (16.6)	
Liver met				
No	388 (82.6)	100 (75.2)	488 (80.9)	0.074
Yes	82 (17.4)	33 (24.8)	115 (19.1)	
Line of treatment				
First	329 (69.6)	74 (54.4)	403 (66.2)	0.001
Subsequent	144 (30.4)	62 (45.6)	206 (33.8)	
CT-ICI	125 (26.4)	32 (23.5)	157 (25.8)	0.191
ICI-ICI	11 (2.3)	2 (1.5)	13 (2.1)	
ICI	325 (68.7)	102 (75.0)	427 (70.1)	
immuno-TKI	12 (2.5)		12 (2.0)	
dNLR				
Mean (SD)	2.6 (2.0)	4.2 (2.8)	3.0 (2.3)	< 0.001
LIPI				
0	203 (55.6)	14 (14.1)	217 (46.8)	< 0.001
1	130 (35.6)	51 (51.5)	181 (39.0)	
2	32 (8.8)	34 (34.3)	66 (14.2)	

*ECOG PS* Eastern Cooperative Oncology Group performance status, *NSCLC* non-small cell lung cancer, *Num.* number, *met*. Metastasis, *ICI* immune checkpoint inhibitor, *CT* chemotherapy, *TKI* tyrosine kinase inhibitor, *dNLR* derived neutrophil-to-lymphocyte ratio, *LIPI* lung immune prognostic index

Fifty-four patients (8.4%) died within 30 days. The distribution of baseline features according to 90-day mortality is reported in Table S3.

In total, 220 patients (34.5%) had disease progression or death within 90 days. The distribution of baseline features according to early progression is reported in Table S4.

At univariable analyses, patients with ECOG PS 2, high/intermediate LIPI score, > 3 metastatic sites, brain and lung metastasis, and those treated with a subsequent line of treatment presented an increased risk of 90-day mortality (Table 3). At multivariable analysis, ECOG PS 2 (OR 2.70, p 0.019), high (OR 11.47, p < 0.001), and intermediate LIPI score (OR 4.97, p < 0.001) were independently associated with an increased risk of 90-day mortality (Table 3). To provide further support for the utilization of the LIPI score over the NLR, we assessed the risk of 90-day mortality associated with the NLR, obtaining a less impacting OR of 1.05 (p = 0.002), even if no significant differences were found between the comparison of respective ROC curves (AUC 0.73 vs 0.74, p = 0.21). In addition, NLR prognostic relevance for 90-day mortality was not confirmed within the same multivariable assessment (OR 1.02, p 0.14).

**Table 3:** Univariate and multivariate logistic regression analyses for 90-day mortality in the training cohort

90-day mortality						
Predictors	Univariate			Multivariate		
	Odds Ratios	CI (95%)	p value	Odds Ratios	CI (95%)	p value
Intercept				0.01	0.00–0.14	**0.001**
Age > 65	1.29	0.86–1.93	0.219	0.86	0.44–1.67	0.658
ECOG PS 2	4.28	2.58–7.10	** < 0.001**	2.70	1.18–6.18	**0.019**
High LIPI	15.33	7.42–31.67	** < 0.001**	11.47	4.34–30.31	** < 0.001**
Intermediate LIPI	5.66	3.01–10.64	** < 0.001**	4.97	2.16–11.41	** < 0.001**
Genitourinary cancers	2.34	0.24–22.73	0.462	1.41	0.10–20.08	0.799
Head–neck cancers	4.00	0.48–33.08	0.198	4.71	0.31–70.47	0.262
Melanoma	2.28	0.28–18.57	0.442	1.51	0.13–17.88	0.742
NSCLC	6.48	0.85–49.31	0.071	7.65	0.53–110.94	0.136
ICI	1.37	0.89–2.12	0.155	1.89	0.88–4.09	0.104
Subsequent line of treatment	1.91	1.29–2.82	**0.001**	4.43	0.85–23.02	0.077
> 3 metastatic sites	2.35	1.32–4.17	**0.004**	0.85	0.34–2.16	0.735
Brain met	1.87	1.17–3.01	**0.009**	1.80	0.77–4.21	0.172
Liver met	1.56	0.98–2.47	0.059	1.00	0.41–2.45	0.995
Lung met	1.73	1.16–2.58	**0.007**	1.79	0.88–3.66	0.108

*CI* confidence interval, *ECOG PS* Eastern Cooperative Oncology Group performance status, *LIPI* lung immune prognostic index, *NSCLC* non-small cell lung cancer, *ICI* immune checkpoint inhibitor, *met*., metastasis

At univariable analyses, patients with ECOG PS 2, high/intermediate LIPI score, lung and liver metastasis, and those treated with a subsequent line of treatment presented an increased risk of 30-day mortality (Table S5). At multivariable analysis, lung metastasis (OR 2.66, p = 0.048), high (OR 8.09, p = 0.006) and intermediate LIPI score (OR 8.62, p = 0.001) were independently associated with increased risk of 30-day mortality (Table S5).

At univariable analyses, patients with ECOG PS 2, high LIPI score, NSCLC histology, > 3 metastatic sites, lung metastasis, and those treated with ICI single agent and a subsequent line of treatment presented an increased risk of early progression (Table S6). At multivariable analysis, high (OR 8.11, p < 0.001) and intermediate (OR 2.63, p = 0.002) LIPI scores were independently associated with increased risk of early progression (Table S6).

### Nomogram for 90-day mortality

Next, we sought to build a nomogram to predict 90-day mortality using the variables that were significantly associated with increased risk of death at 90 days in the multivariable model. Among 637 patients in the training cohort, 212 were excluded because of missing ECOG PS or LIPI data or because they were alive with a follow-up < 3 months, for a total of 425 patients included in the analysis.

**Fig. 1:**
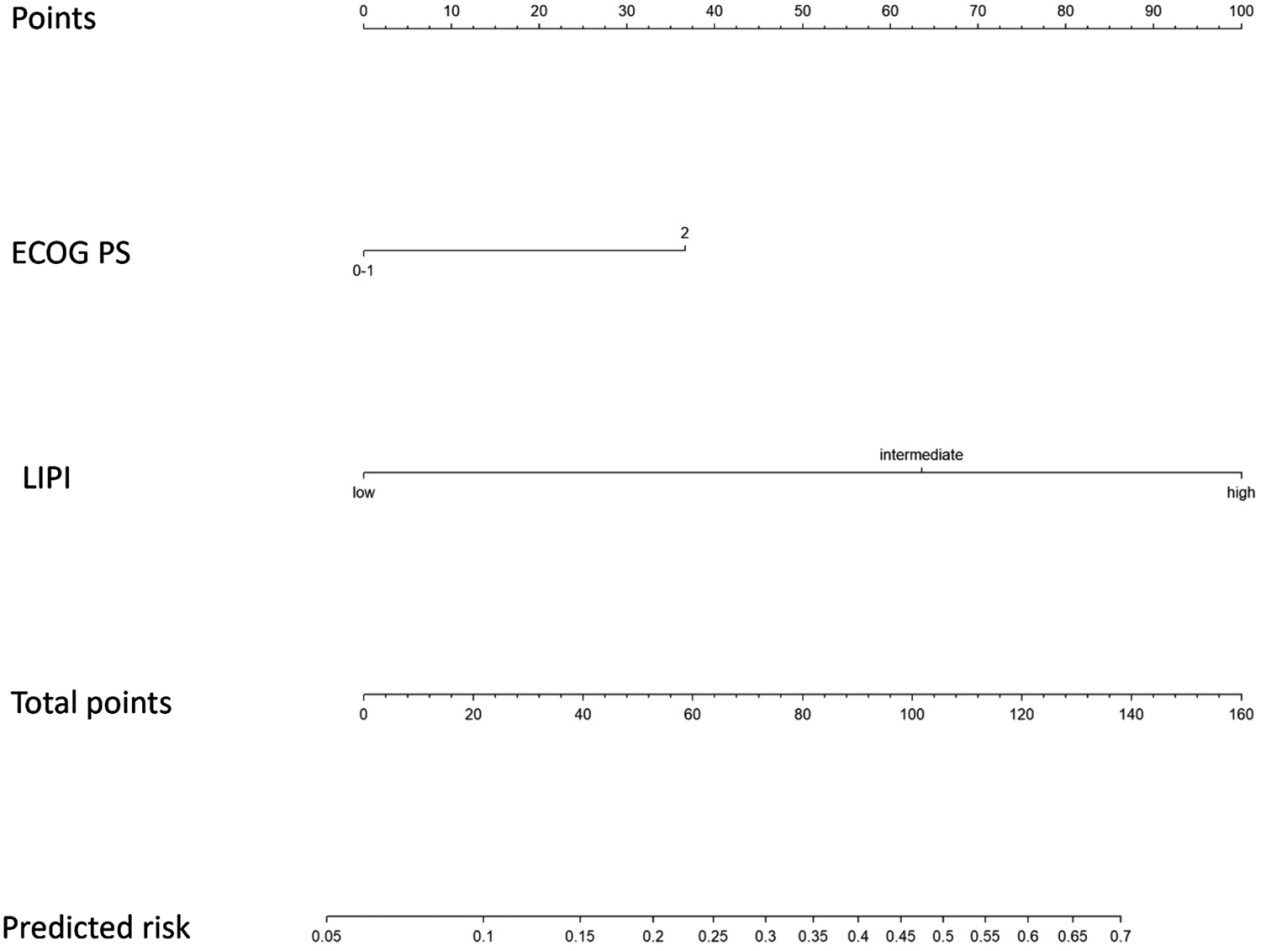
Nomogram for 90-day mortality prediction. ECOG PS, Eastern Cooperative Oncology Group performance status; LIPI, lung immune prognostic index

**Fig. 2:**
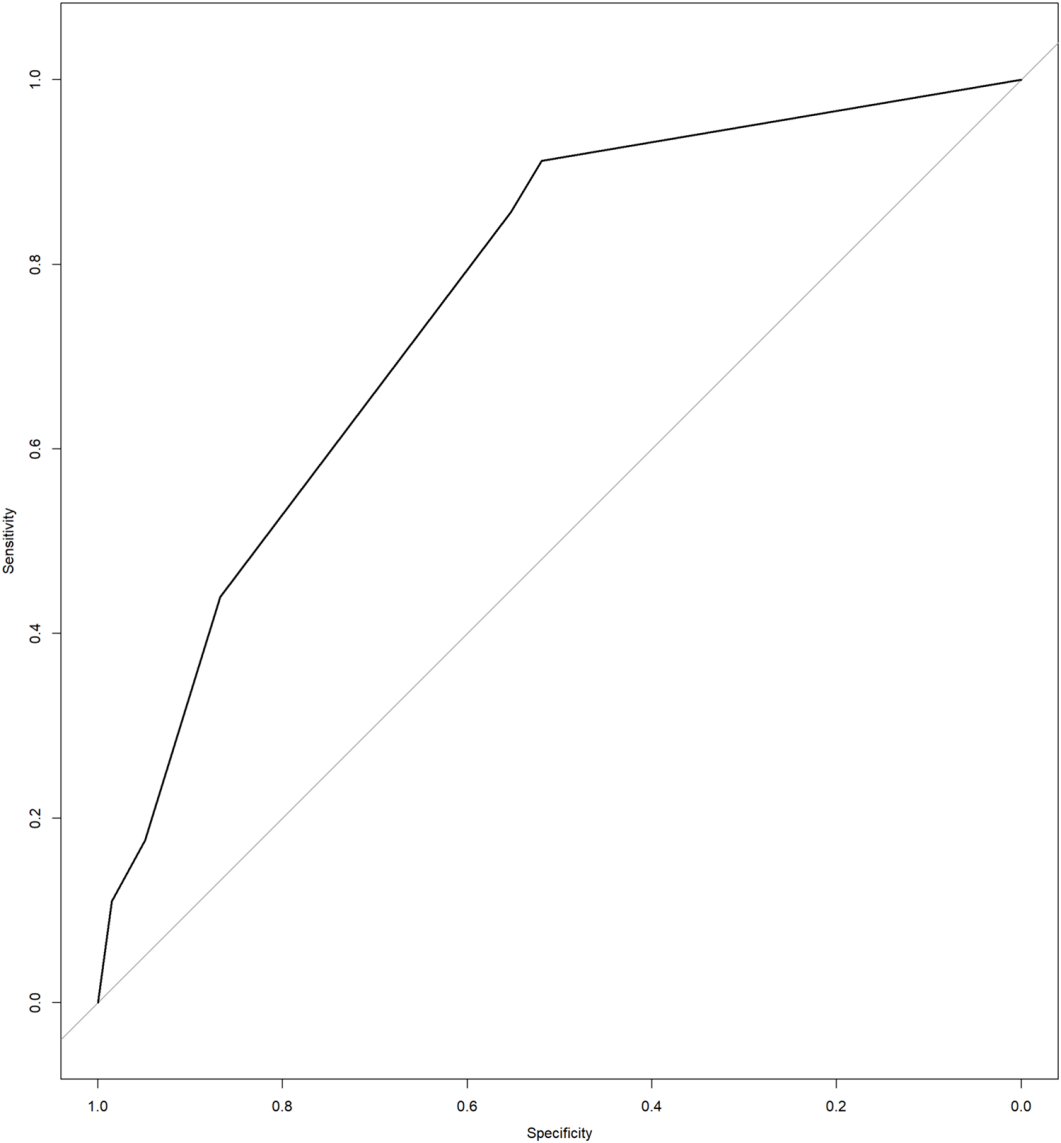
Receiver operating characteristic (ROC) curve for 90-day mortality prediction

Based on the multivariable assessment, the produced score confers 37 points for ECOG PS 2, 64 points for an intermediate LIPI score, and 100 points for a high LIPI score. Patients with the maximum score (137) had a 70% risk of death within 90 days from treatment start. A nomogram representing the model is provided in Fig. 1. The area under the ROC for the score was 0.76 (95% CI 0.71–0.81) for 90-day mortality prediction (Fig. 2) with a concordance index of 0.76. The same analysis was performed within each histology group. The AUC was 0.73 (95%CI, 0.67–0.79) for NSCLC, 0.85 (95%CI, 0.73–0.96) for melanoma, and 0.78 (95%CI, 0.67–0.89) for other tumor types (GU, GI, head–neck).

### Validation cohort

A total of 255 patients were included in the validation cohort. Most patients were males (66.7%) and had an ECOG PS of 0–1 (92.2%). NSCLC was the most frequent tumor type (67.5%), followed by genitourinary (23.5%) and melanoma (9%). CT-ICI combination and ICI monotherapy were the most frequently used regimens (43.9% and 40%, respectively) and the majority of patients (74.9%) were treated in first line (Table S7). Of them, 37.3% were in the low-risk group (n = 95), 41.6% in the intermediate-risk group (n = 106), and 21.2% in the high-risk group (n = 54). After a median follow-up of 27.8 months (95%CI, 23.9–31.3), 106 patients were alive (41.6%), and the median OS was 15.5 months (95% CI 12.5–22.3).

Overall, 46 patients (18%) died within 90 days. The baseline characteristics according to 90-day mortality are reported in Table S8. The multivariable regression analysis for 90-day mortality risk is given in Table S9.

When the score for 90-day mortality was applied, the area under the ROC was 0.72 (95% CI 0.64–0.80), p < 0.001.

## Discussion

We conducted a study on a cohort of 637 patients with advanced solid tumors treated with ICI, either as single agent or in combination with chemotherapy or other drugs. Our findings showed that 21.4% and 8.8% of patients died within 90 days and 30 days from treatment start, respectively. Furthermore, 35.3% of patients experienced early progression (≤ 90 days) of their disease. We also analyzed the laboratory and clinical factors that contributed to EM and found that LIPI score and ECOG PS were independent predictors of 90-day mortality as a primary objective. Results from the study population were externally validated in 255 patients, and the prognostic role of intermediate–high LIPI score was confirmed.

Based on our results, we developed a novel scoring system that can predict 90-day mortality with a good degree of accuracy (AUC of 0.76), which was further confirmed in the external validation cohort (AUC of 0.72).

The definition of EM ranges from 30 to 90 days after the start of treatment, with a prevalence of 20 to 35%, depending on the type of treatment and disease burden [12, 13]. In the context of "desperation oncology," a high likelihood of 30-day mortality may compromise the pursuit of curative treatment. Conversely, the use of a 90-day cutoff could assist in tailoring the intensity of treatment, particularly when considering various initial therapeutic options.

According to a recent meta-analysis of 56 randomized controlled trials involving over 40,000 patients with various solid cancers, the rate of early death (≤ 90 days) was higher with single-agent ICI treatment compared to other ICI treatments (14.2% vs. 6.7%) [14]. Our findings from a real-world context evidenced a slightly increased early death rate (21.4%) compared with their results, with no difference according to treatment received.

A large cohort study investigated the cause of EM, defined as within 60 days from treatment initiation among 7126 patients affected by solid cancers treated with ICI [4]. NSCLC was the predominant tumor type (58.1%), followed by melanoma (23.3%) and other tumors, reflecting the epidemiology of our cohort [4]. Noteworthy, only 37.7% of patients had a stage IV disease at diagnosis, receiving predominantly ICI alone (57.8%). Patients treated at tertiary centers, those admitted to the hospital and treated with prior radiation therapy or chemotherapy had the greater adjusted probability of 60-day mortality, as well as those who presented higher Edmonton Symptom Assessment System (ESAS) scores, anemia, and leukocytosis [4]. Conversely, patients presenting low NLR or higher BMI, and those receiving ICI + ICI had a lower risk of 60-day mortality [4]. Interestingly, this large study evidenced a prognostic role of clinical conditions and laboratory tests, suggesting an external validity of our findings relative to LIPI and ECOG PS values.

Prescribing immunotherapy to frail patients may be influenced by an overestimation of the potential benefits of novel therapies or inadequate evaluation of deteriorating clinical conditions, even if for treatment-naïve or young patients. Remarkably, individuals with a baseline ECOG PS 2 or higher were associated with reduced survival rates and a higher probability of receiving ICI during the last month of life [15–17]. Furthermore, no efficacy differences were found between ECOG PS 0 or 1 in solid cancer patients under ICI-based regimens in a comprehensive meta-analysis [18], confirming the discriminative importance of ECOG PS 2.

On the other hand, ECOG PS may not be informative enough and be connected to comorbidities or to specific cancer-related symptoms that may benefit from anticancer treatments themselves. Notwithstanding efficacy reduction, prospective trials on NSCLC patients confirmed that single-agent ICI may exhibit an acceptable toxicity profile for frail patients, paving the way for prescription [19, 20]

The use of laboratory values may be useful in this setting to further select patients. In this context, the LIPI score, as previously described, combines dNLR and LDH levels with an established prognostic validity under immunotherapy regardless of the setting of treatment and disease type, reflecting an inflammatory status of the organism [7, 9]. In addition, incrementable dNLR has been validated as a surrogate marker of pro-tumor tumor microenvironment (TME) composition in a retrospective observational work including 221 advanced NSCLC patients treated with upfront pembrolizumab [21]. In particular, low dNLR (< 2.6) was associated with higher numbers of tumor-associated CD8 + , FOXP3 + , PD-1 + immune cells within TME [21]. Indeed, altered NLR and LDH have been associated with EM [4, 6] or HPD [22, 23] in several experiences. It should be noted that these findings may not be generalizable due to the lack of data on other types than NSCLC and the limitation of analyses focusing on EM. Even if we did not find any differences between LIPI and NLR ROC curves, we justify the use of a composite score considering the almost tripled risk of EM for the LIPI high risk in comparison with the intermediate group and the loss of significance within the multivariable assessment for NLR alone.

In an observational work performed by our research team, the short-term prognostic value of the LIPI score was investigated for the first time among advanced NSCLC patients treated with single-agent immunotherapy [23]. An intermediate–high LIPI score was independently associated with increased 90-day mortality risk. Notably, we confirmed the superiority of a combined clinical–laboratory test score, such as the modified palliative prognostic (PaP) score that includes performance status, pivotal clinical symptoms (dyspnea, anorexia), and total leukocyte and lymphocyte counts [24].

After an internal validation of the prognostic relevance of ECOG PS and LIPI scores regardless of malignancies and type of treatment, we developed and externally validated a 90-day prognostic score with a good capability of early mortality risk assessment in the present work.

In addition, we confirmed that an intermediate–high LIPI score was an independent risk factor for 30-day mortality and early progression (≤ 90 days).

However, it is crucial to note that our study has some limitations. Firstly, it is a retrospective analysis, and further larger prospective studies are required to validate our findings. The rate of missing data for certain variables, such as LIPI, was consistent, which could lead to selection bias.

Secondly, our study population consisted primarily of patients with lung cancer, which may limit the generalizability of our results to other cancer types. Finally, no central revision of radiological imaging has been assessed, limiting the findings about radiological progression.

Despite these limitations, our study highlights the importance of EM prediction and personalized treatment strategies for advanced cancer patients. Moreover, this is the first study investigating the short-term prognostic value of LIPI, including patients of multiple malignancies (NSCLC, melanoma, head–neck, others) treated with single-agent ICIs but also ICI combinations (chemotherapy, other ICI, TKI), and the score developed is easily performable with a good performance in an external cohort.

In situations with a high probability of EM (137 points), this nomogram can assist in determining the appropriate level of treatment when multiple treatment options are available, including the addition of chemotherapy. Combination strategies have been shown to provide short-term benefits for advanced NSCLC [25], and this approach can also be applied to other types of cancer [14]. Our nomogram can aid healthcare providers in cases where available biomarkers, such as PD-1 expression, may not provide sufficient guidance.

## Conclusion

In conclusion, our study emphasizes that the LIPI score and ECOG PS are independent predictors of 90-day mortality in the internal cohort. Importantly, the LIPI score also demonstrates significant prognostic value for 30-day mortality and early progression, making it a valuable tool for stratifying patients in clinical research and daily practice. Furthermore, the short-term prognostic significance of the LIPI score remains consistent in the validation cohort, underscoring its broad applicability in clinical practice regardless of the type of ICI-based regimen used. Our nomogram can assist clinicians in identifying patients at high risk of EM.

## Supplementary Information

Below is the link to the electronic supplementary material.

Supplementary file 1 (DOCX 47 KB)

## Data Availability

The data underlying this article cannot be shared due to the privacy of individuals who participated in the study, as stated by the local Ethics Committee (approval no. 2381/2019). Additional aggregated data analyses and the underlying analytic R code are available from the authors upon request.
