# Supplementary material for: Development and validation of a new tool to estimate early mortality in patients with advanced cancer treated with immunotherapy
Source: Cancer Immunol Immunother. 2024 Oct 3;73(12):246. doi: 10.1007/s00262-024-03836-w (PMC11447166; doi:10.1007/s00262-024-03836-w)
Supplement: Supplementary file 1 — (DOCX 47 KB) [file 262_2024_3836_MOESM1_ESM.docx]

| Overall survival | | | | |
| --- | --- | --- | --- | --- |
|  |  | all | HR (univariable) | HR (multivariable) |
|  |  |  |  |  |
| Age | >65 | 398 (62.6) | 1.20 (0.97-1.48, p=0.088) | 1.35 (0.97-1.87, p=0.073) |
|  | ≤65 | 238 (37.4) | - | - |
| ECOG PS | 0-1 | 511 (86.8) | - | - |
|  | 2 | 78 (13.2) | 2.63 (2.01-3.45, p<0.001) | 2.09 (1.39-3.14, p<0.001) |
| LIPI | low | 230 (47.0) | - | - |
|  | high | 71 (14.5) | 4.92 (3.54-6.84, p<0.001) | 3.50 (2.26-5.42, p<0.001) |
|  | intermediate | 188 (38.4) | 2.06 (1.58-2.68, p<0.001) | 1.55 (1.08-2.22, p=0.017) |
| Histology | Gastrointestinal | 20 (3.1) | - | - |
|  | Genitourinary | 41 (6.4) | 0.80 (0.30-2.09, p=0.645) | 1.31 (0.43-4.02, p=0.641) |
|  | Head-neck | 72 (11.3) | 2.06 (0.92-4.63, p=0.079) | 2.82 (0.97-8.20, p=0.057) |
|  | Melanoma | 110 (17.3) | 1.27 (0.58-2.77, p=0.544) | 1.88 (0.69-5.14, p=0.217) |
|  | NSCLC | 393 (61.8) | 2.35 (1.11-4.97, p=0.026) | 2.65 (0.92-7.65, p=0.072) |
| Type of treatment | ICI-other | 435 (68.4) | - | - |
|  | ICI | 201 (31.6) | 0.84 (0.67-1.06, p=0.144) | 1.01 (0.69-1.47, p=0.971) |
| Line of treatment | First | 426 (67.0) | - | - |
|  | Subsequent | 210 (33.0) | 1.77 (1.45-2.16, p<0.001) | 1.72 (0.95-3.11, p=0.071) |
| Num. of metastatic sites | >3 | 76 (16.3) | 1.54 (1.13-2.11, p=0.007) | 0.89 (0.55-1.44, p=0.639) |
|  | ≤3 | 391 (83.7) | - | - |
| Brain met. | no | 526 (83.4) | - | - |
|  | yes | 105 (16.6) | 1.64 (1.27-2.10, p<0.001) | 1.32 (0.87-2.00, p=0.198) |
| Liver met. | no | 514 (81.6) | - | - |
|  | yes | 116 (18.4) | 1.32 (1.04-1.68, p=0.022) | 1.52 (1.00-2.30, p=0.048) |
| Lung met. | no | 291 (46.2) | - | - |
|  | yes | 339 (53.8) | 1.35 (1.10-1.65, p=0.004) | 1.10 (0.79-1.52, p=0.584) |

Table 1S. Univariate and multivariate Cox regression analyses for overall survival. Abbreviations: HR, hazard ratio; ECOG PS, Eastern Cooperative Oncology Group performance status; NSCLC, non-small cell lung cancer; Num., number; met., metastasis; ICI, immune-checkpoint inhibitor; LIPI, lung immune-prognostic index.

| Progression-free survival | | | | |
| --- | --- | --- | --- | --- |
|  |  | all | HR (univariable) | HR (multivariable) |
| Age | >65 | 398 (62.6) | 1.15 (0.96-1.38, p=0.134) | 1.23 (0.94-1.62, p=0.135) |
|  | ≤65 | 238 (37.4) | - | - |
| ECOG PS | 0-1 | 511 (86.8) | - | - |
|  | 2 | 78 (13.2) | 1.95 (1.51-2.51, p<0.001) | 1.29 (0.88-1.89, p=0.191) |
| LIPI | low | 230 (47.0) | - | - |
|  | high | 71 (14.5) | 3.62 (2.67-4.89, p<0.001) | 2.97 (2.01-4.39, p<0.001) |
|  | intermediate | 188 (38.4) | 1.93 (1.54-2.42, p<0.001) | 1.68 (1.25-2.26, p=0.001) |
| Histology | Gastrointestinal | 20 (3.1) | - | - |
|  | Genitourinary | 41 (6.4) | 1.12 (0.48-2.65, p=0.794) | 1.83 (0.68-4.92, p=0.234) |
|  | Head-neck | 72 (11.3) | 2.39 (1.12-5.10, p=0.024) | 3.66 (1.35-9.94, p=0.011) |
|  | Melanoma | 110 (17.3) | 1.39 (0.67-2.89, p=0.374) | 1.78 (0.70-4.51, p=0.223) |
|  | NSCLC | 393 (61.8) | 3.20 (1.59-6.46, p=0.001) | 4.71 (1.80-12.34, p=0.002) |
| Type of treatment | ICI-other | 435 (68.4) | - | - |
|  | ICI | 201 (31.6) | 0.97 (0.79-1.19, p=0.775) | 0.90 (0.66-1.23, p=0.518) |
| Line of treatment | First | 426 (67.0) | - | - |
|  | Subsequent | 210 (33.0) | 1.50 (1.26-1.80, p<0.001) | 1.76 (0.98-3.14, p=0.057) |
| Num. of metastatic sites | >3 | 76 (16.3) | 1.43 (1.09-1.88, p=0.010) | 0.87 (0.57-1.32, p=0.506) |
|  | ≤3 | 391 (83.7) | - | - |
| Brain met. | no | 526 (83.4) | - | - |
|  | yes | 105 (16.6) | 1.48 (1.18-1.86, p=0.001) | 1.16 (0.80-1.67, p=0.441) |
| Liver met. | no | 514 (81.6) | - | - |
|  | yes | 116 (18.4) | 1.25 (1.01-1.56, p=0.044) | 1.67 (1.16-2.41, p=0.006) |
| Lung met. | no | 291 (46.2) | - | - |
|  | yes | 339 (53.8) | 1.38 (1.15-1.65, p<0.001) | 1.23 (0.93-1.62, p=0.148) |

Table 2S. Univariate and multivariate Cox regression analyses for progression-free survival. Abbreviations: HR, hazard ratio; ECOG PS, Eastern Cooperative Oncology Group performance status; NSCLC, non-small cell lung cancer; Num., number; met., metastasis; ICI, immune-checkpoint inhibitor; LIPI, lung immune-prognostic index.

|  |  | 30-day mortality  No  (%) | 30-day mortality  Yes  (%) | Total  (%) | p  value |
| --- | --- | --- | --- | --- | --- |
| Age | >65 | 355 (62.6) | 36 (66.7) | 391 (63.0) | 0.658 |
|  | ≤65 | 212 (37.4) | 18 (33.3) | 230 (37.0) |  |
| Sex | Female | 218 (38.5) | 20 (37.0) | 238 (38.4) | 0.947 |
|  | Male | 348 (61.5) | 34 (63.0) | 382 (61.6) |  |
| ECOG PS | 0-1 | 463 (88.4) | 34 (68.0) | 497 (86.6) | <0.001 |
|  | 2 | 61 (11.6) | 16 (32.0) | 77 (13.4) |  |
| Smoking history | current smoker | 99 (24.7) | 10 (21.3) | 109 (24.3) | 0.250 |
|  | former smoker | 245 (61.1) | 26 (55.3) | 271 (60.5) |  |
|  | never smoker | 57 (14.2) | 11 (23.4) | 68 (15.2) |  |
| Histology | Gastrointestinal | 18 (3.2) | 1 (1.9) | 19 (3.1) | 0.053 |
|  | Genitourinary | 33 (5.8) | 1 (1.9) | 34 (5.5) |  |
|  | Head-neck | 63 (11.1) | 6 (11.1) | 69 (11.1) |  |
|  | Melanoma | 107 (18.9) | 3 (5.6) | 110 (17.7) |  |
|  | NSCLC | 346 (61.0) | 43 (79.6) | 389 (62.6) |  |
| Num. of metastatic sites | >3 | 67 (15.9) | 7 (22.6) | 74 (16.4) | 0.474 |
|  | ≤3 | 354 (84.1) | 24 (77.4) | 378 (83.6) |  |
| Lung met. | no | 265 (47.2) | 15 (27.8) | 280 (45.5) | 0.009 |
|  | yes | 296 (52.8) | 39 (72.2) | 335 (54.5) |  |
| Brain met. | no | 471 (83.8) | 43 (79.6) | 514 (83.4) | 0.550 |
|  | yes | 91 (16.2) | 11 (20.4) | 102 (16.6) |  |
| Liver met. | no | 461 (82.2) | 38 (70.4) | 499 (81.1) | 0.053 |
|  | yes | 100 (17.8) | 16 (29.6) | 116 (18.9) |  |
| Line of treatment | First | 387 (68.3) | 27 (50.0) | 414 (66.7) | 0.010 |
|  | Subsequent | 180 (31.7) | 27 (50.0) | 207 (33.3) |  |
| Type of treatment | CT-ICI | 390 (68.8) | 42 (77.8) | 432 (69.6) | 0.223 |
|  | ICI-ICI | 177 (31.2) | 12 (22.2) | 189 (30.4) |  |
|  | ICI | 2.8 (2.1) | 4.5 (3.2) | 3.0 (2.3) | <0.001 |
|  | immuno-TKI | 219 (50.6) | 5 (11.9) | 224 (47.2) | <0.001 |
| dLNR | Mean (SD) | 159 (36.7) | 23 (54.8) | 182 (38.3) |  |
| lipi | 0 | 55 (12.7) | 14 (33.3) | 69 (14.5) |  |

Table 3S. Baseline characteristics according to 30-day mortality. Abbreviations: ECOG PS, Eastern Cooperative Oncology Group performance status; NSCLC, non-small cell lung cancer; Num., number; met., metastasis; ICI, immune-checkpoint inhibitor; CT, chemotherapy; TKI, tyrosine-kinase inhibitor; dNLR, derived neutrophil-to-lymphocyte ratio; LIPI, lung immune-prognostic index.

|  |  | 90-day progression  No  (%) | 90-day progression  Yes  (%) | Total  (%) | p  value |
| --- | --- | --- | --- | --- | --- |
| Age | >65 | 244 (62.7) | 141 (64.1) | 385 (63.2) | 0.804 |
|  | ≤65 | 145 (37.3) | 79 (35.9) | 224 (36.8) |  |
| Sex | Female | 151 (38.8) | 82 (37.4) | 233 (38.3) | 0.804 |
|  | Male | 238 (61.2) | 137 (62.6) | 375 (61.7) |  |
| ECOG PS | 0-1 | 329 (91.9) | 158 (77.5) | 487 (86.7) | <0.001 |
|  | 2 | 29 (8.1) | 46 (22.5) | 75 (13.3) |  |
| Smoking history | current smoker | 63 (24.7) | 44 (24.0) | 107 (24.4) | 0.984 |
|  | former smoker | 154 (60.4) | 112 (61.2) | 266 (60.7) |  |
|  | never smoker | 38 (14.9) | 27 (14.8) | 65 (14.8) |  |
| Histology | Gastrointestinal | 16 (4.1) | 2 (0.9) | 18 (3.0) | <0.001 |
|  | Genitourinary | 25 (6.4) | 8 (3.6) | 33 (5.4) |  |
|  | Head-neck | 43 (11.1) | 20 (9.1) | 63 (10.3) |  |
|  | Melanoma | 90 (23.1) | 20 (9.1) | 110 (18.1) |  |
|  | NSCLC | 215 (55.3) | 170 (77.3) | 385 (63.2) |  |
| Num. of metastatic sites | >3 | 44 (14.1) | 29 (22.7) | 73 (16.6) | 0.040 |
|  | ≤3 | 268 (85.9) | 99 (77.3) | 367 (83.4) |  |
| Lung met. | no | 192 (49.6) | 78 (36.1) | 270 (44.8) | 0.002 |
|  | yes | 195 (50.4) | 138 (63.9) | 333 (55.2) |  |
| Brain met. | no | 331 (85.3) | 173 (80.1) | 504 (83.4) | 0.124 |
|  | yes | 57 (14.7) | 43 (19.9) | 100 (16.6) |  |
| Liver met. | no | 322 (83.2) | 166 (76.9) | 488 (80.9) | 0.073 |
|  | yes | 65 (16.8) | 50 (23.1) | 115 (19.1) |  |
| Line of treatment | First | 282 (72.5) | 120 (54.5) | 402 (66.0) | <0.001 |
|  | Subsequent | 107 (27.5) | 100 (45.5) | 207 (34.0) |  |
| Type of treatment | CT-ICI | 262 (67.4) | 165 (75.0) | 427 (70.1) | 0.059 |
|  | ICI-ICI | 127 (32.6) | 55 (25.0) | 182 (29.9) |  |
|  | ICI | 2.5 (1.9) | 3.8 (2.7) | 3.0 (2.3) | <0.001 |
|  | immuno-TKI | 177 (58.6) | 39 (23.9) | 216 (46.5) | <0.001 |
| dLNR | Mean (SD) | 105 (34.8) | 77 (47.2) | 182 (39.1) |  |
| lipi | 0 | 20 (6.6) | 47 (28.8) | 67 (14.4) |  |

Table 4S. Baseline characteristics according to 90-day progression. Abbreviations: ECOG PS, Eastern Cooperative Oncology Group performance status; NSCLC, non-small cell lung cancer; Num., number; met., metastasis; ICI, immune-checkpoint inhibitor; CT, chemotherapy; TKI, tyrosine-kinase inhibitor; dNLR, derived neutrophil-to-lymphocyte ratio; LIPI, lung immune-prognostic index.

| **30-day mortality** | | | | | | |
| --- | --- | --- | --- | --- | --- | --- |
|  |  | **Univariate** |  | **Multivariate** | | |
| *Predictors* | *Odds*  *Ratios* | *CI (95%)* | *p value* | *Odds Ratios* | *CI* | *p value* |
| Intercept |  |  |  | 0.00 | 0.00 – 0.09 | **0.001** |
| Age >65 | 1.20 | 0.66 – 2.16 | 0.551 | 0.85 | 0.36 – 2.03 | 0.722 |
| ECOG PS 2 | 3.65 | 1.90 – 7.02 | **<0.001** | 2.20 | 0.77 – 6.28 | 0.139 |
| High LIPI | 11.41 | 3.94 – 33.05 | **<0.001** | 8.09 | 1.82 – 35.98 | **0.006** |
| Intermediate LIPI | 6.29 | 2.34 – 16.89 | **<0.001** | 8.62 | 2.38 – 31.23 | **0.001** |
| Genitourinary cancers | 0.49 | 0.03 – 8.23 | 0.618 | 0.34 | 0.02 – 7.50 | 0.493 |
| Head-neck cancers | 1.77 | 0.20 – 15.68 | 0.608 | 3.28 | 0.14 – 77.46 | 0.462 |
| Melanoma | 0.50 | 0.05 – 5.12 | 0.563 | 0.55 | 0.04 – 8.49 | 0.668 |
| NSCLC | 2.24 | 0.29 – 17.18 | 0.439 | 5.12 | 0.21 – 122.31 | 0.313 |
| ICI single-agent | 1.59 | 0.82 – 3.10 | 0.170 | 1.94 | 0.69 – 5.41 | 0.206 |
| Subsequent line of treatment | 2.14 | 1.22 – 3.76 | **0.008** | 9.05 | 0.84 – 98.00 | 0.070 |
| >3 metastatic sites | 1.55 | 0.64 – 3.74 | 0.330 | 0.58 | 0.17 – 1.94 | 0.378 |
| Brain met. | 1.31 | 0.65 – 2.64 | 0.446 | 1.37 | 0.43 – 4.35 | 0.597 |
| Liver met. | 1.95 | 1.05 – 3.63 | **0.036** | 1.35 | 0.45 – 4.07 | 0.589 |
| Lung met. | 2.31 | 1.25 – 4.29 | **0.008** | 2.66 | 1.01 – 7.02 | **0.048** |
|  |  |  |  |  | | |
|  |  |  |  |  | | |

Table 5S. Univariate and multivariate logistic regression analyses for 30-day mortality. Abbreviations: CI, confidence interval; ECOG PS, Eastern Cooperative Oncology Group performance status; LIPI, lung immune-prognostic index; NSCLC, non-small cell lung cancer; ICI, immune-checkpoint inhibitor; met., metastasis.

| **90-day progression** | | | | | | |
| --- | --- | --- | --- | --- | --- | --- |
|  |  | **Univariate** |  | **Multivariate** | | |
| *Predictors* | *Odds*  *Ratios* | *CI (95%)* | *p value* | *Odds Ratios* | *CI* | *p value* |
| Intercept |  |  |  | 0.05 | 0.01 – 0.35 | **0.002** |
| Age >65 | 1.06 | 0.75 – 1.50 | 0.737 | 0.81 | 0.47 – 1.41 | 0.464 |
| ECOG PS 2 | 3.30 | 2.00 – 5.46 | **<0.001** | 1.88 | 0.87 – 4.09 | 0.109 |
| High LIPI | 11.41 | 3.94 – 33.05 | **<0.001** | 8.11 | 3.70 – 17.77 | **<0.001** |
| Intermediate LIPI | 3.33 | 2.11 – 5.24 | <0.001 | 2.63 | 1.44 – 4.81 | **0.002** |
| Genitourinary cancers | 2.56 | 0.48 – 13.62 | 0.270 | 2.62 | 0.39 – 17.51 | 0.319 |
| Head-neck cancers | 3.72 | 0.78 – 17.76 | 0.099 | 3.27 | 0.47 – 22.61 | 0.229 |
| Melanoma | 1.78 | 0.38 – 8.36 | 0.466 | 1.09 | 0.17 – 6.95 | 0.924 |
| NSCLC | 6.33 | 1.43 – 27.89 | **0.015** | 4.94 | 0.75 – 32.35 | 0.096 |
| ICI single-agent | 1.45 | 1.00 – 2.11 | **0.048** | 1.58 | 0.84 – 2.96 | 0.152 |
| Subsequent line of treatment | 2.20 | 1.55 – 3.11 | **<0.001** | 2.05 | 0.62 – 6.81 | 0.240 |
| >3 metastatic sites | 1.78 | 1.06 – 3.01 | **0.030** | 0.91 | 0.40 – 2.07 | 0.820 |
| Brain met. | 1.44 | 0.93 – 2.23 | 0.099 | 1.20 | 0.57 – 2.55 | 0.633 |
| Liver met. | 1.49 | 0.99 – 2.26 | 0.058 | 1.38 | 0.66 – 2.88 | 0.399 |
| Lung met. | 1.74 | 1.24 – 2.45 | **0.001** | 1.47 | 0.83 – 2.61 | 0.190 |
|  |  |  |  |  | | |
|  |  |  |  |  | | |

Table 6S. Univariate and multivariate logistic regression analyses for 90-day progression. Abbreviations: CI, confidence interval; ECOG PS, Eastern Cooperative Oncology Group performance status; LIPI, lung immune-prognostic index; NSCLC, non-small cell lung cancer; ICI, immune-checkpoint inhibitor; met., metastasis.

|  | **Overall (N=255)** |
| --- | --- |
| **Age** |  |
| ≤65 | 96 (37.6%) |
| >65 | 159 (62.4%) |
| **Sex** |  |
| Female | 85 (33.3%) |
| Male | 170 (66.7%) |
| **ECOG PS** |  |
| 0-1 | 235 (92.2%) |
| 2 | 20 (7.8%) |
| **Smoking status** |  |
| current smoker | 69 (27.1%) |
| former smoker | 88 (34.5%) |
| never smoker | 12 (4.7%) |
| Missing | 86 (33.7%) |
| **Histology** |  |
| Genitourinary | 60 (23.5%) |
| Melanoma | 23 (9%) |
| NSCLC | 172 (67.5%) |
| **Num. of metastatic**  **sites** |  |
| ≤3 | 181 (71%) |
| >3 | 74 (29%) |
| **Lung met.** |  |
| no | 74 (29%) |
| yes | 181 (71%) |
| **Brain met.** |  |
| no | 193 (75.7%) |
| yes | 62 (24.3%) |
| **Liver met.** |  |
| no | 209 (82%) |
| yes | 46 (18%) |
| **Line of treatment** |  |
| First | 191 (74.9%) |
| Subsequent | 64 (25.1%) |
| **Type of treatment** |  |
| CT-ICI | 112 (43.9%) |
| ICI-ICI | 20 (7.8%) |
| ICI | 102 (40%) |
| immuno-TKI | 21 (8.2%) |
| **dLNR** |  |
| Mean (SD) | 2.5 (2.4) |
| Median [Min, Max] | 2.36 [0.1, 20.4] |
| **LIPI** |  |
| high | 54 (21.2%) |
| intermediate | 106 (41.6%) |
| low | 95 (37.3%) |

Table 7S. Baseline characteristics in the validation cohort. Abbreviations: ECOG PS, Eastern Cooperative Oncology Group performance status; NSCLC, non-small cell lung cancer; Num., number; met., metastasis; ICI, immune-checkpoint inhibitor; CT, chemotherapy; TKI, tyrosine-kinase inhibitor; dNLR, derived neutrophil-to-lymphocyte ratio; LIPI, lung immune-prognostic index.

|  |  | 90-day mortality  No  (%) | 90-day mortality Yes  (%) | p  value |
| --- | --- | --- | --- | --- |
| Age | >65 | 128 (61.2) | 31 (67.4) | 0.503 |
|  | ≤65 | 81 (38.8) | 15 (32.6) |  |
| Sex | Female | 73 (34.9) | 12 (26.1) | 0.301 |
|  | Male | 136 (65.1) | 34 (73.9) |  |
| ECOG PS | 0-1 | 197 (94.3) | 38 (82.6) | **0.014** |
|  | 2 | 12 (5.7) | 8 (17.4) |  |
| Histology | NSCLC | 143 (68.4) | 29 (63) | **0.006** |
|  | Genitourinary | 53 (25.4) | 7 (15.2) |  |
|  | Melanoma | 13 (6.2) | 10 (21.7) |  |
| Num. of metastatic sites | >3 | 48 (23) | 26 (56.5) | **<.001** |
|  | ≤3 | 161 (77) | 20 (43.5) |  |
| Lung met. | no | 66 (31.6) | 8 (17.4) | 0.072 |
|  | yes | 143 (68.4) | 38 (82.6) |  |
| Brain met. | no | 161 (77) | 32 (69.6) | 0.342 |
|  | yes | 48 (23.0) | 14 (30.4) |  |
| Liver met. | no | 176 (84.2) | 33 (71.7) | 0.057 |
|  | yes | 33 (15.8) | 13 (28.3) |  |
| Line of treatment | First | 162 (77.5) | 29 (63) | 0.059 |
|  | Subsequent | 47 (22.5) | 17 (37) |  |
| Type of treatment | CT-ICI | 99 (47.4) | 13 (28.3) | **0.003** |
|  | ICI-ICI | 12 (5.7) | 8 (17.4) |  |
|  | ICI | 78 (37.3) | 24 (52.2) |  |
|  | immuno-TKI | 20 (9.6) | 1 (2.2) |  |
| dLNR | Mean (SD) | 3.02 (2.1) | 4.09 (2.9) | **0.006** |
| LIPI score | Low | 89 (42.6) | 8 (13.0) | **<.001** |
|  | Intermediate | 87 (41.6) | 19 (41.3) |  |
|  | High | 33 (15.8) | 21 (45.7) |  |

Table 8S. Baseline characteristics according to 90-day mortality in validation cohort. Abbreviations: ECOG PS, Eastern Cooperative Oncology Group performance status; NSCLC, non-small cell lung cancer; Num., number; met., metastasis; ICI, immune-checkpoint inhibitor; CT, chemotherapy; TKI, tyrosine-kinase inhibitor; dNLR, derived neutrophil-to-lymphocyte ratio; LIPI, lung immune-prognostic index.

|  | **90-day mortality** | | |
| --- | --- | --- | --- |
| *Predictors* | *Odds Ratios* | *CI* | *p* |
| Intercept | 0.01 | 0.00 – 0.05 | **<.001** |
| Age > 65 | 1.54 | 0.67 – 3.57 | 0.312 |
| ECOG PS 2 | 1.56 | 0.51 – 4.73 | 0.433 |
| High LIPI | 9.86 | 3.07 – 31.62 | **<.001** |
| Intermediate LIPI | 3.38 | 1.10 – 10.46 | **0.034** |
| Genitourinary | 0.44 | 0.13 – 1.50 | 0.190 |
| Melanoma | 9.88 | 2.69 – 36.29 | **<.001** |
| >3 metastatic sites | 4.08 | 1.74– 9.57 | **0.001** |
| ICI-other | 1.71 | 0.69 – 4.24 | 0.243 |
| Subsequent line of treatment | 1.52 | 0.53 – 4.39 | 0.440 |
| Brain met | 0.84 | 0.34 – 2.07 | 0.698 |
| Liver met | 0.99 | 0.38 – 2.57 | 0.987 |
| Lung met | 1.72 | 0.65 – 4.88 | 0.258 |
| Observations | 255 | | |
| R^2^ | 0.256 | | |

Table 9s. Logistic regression analysis for 90-day mortality in the validation cohort. Abbreviations: CI, confidence interval; ECOG PS, Eastern Cooperative Oncology Group performance status; LIPI, lung immune-prognostic index; NSCLC, non-small cell lung cancer; ICI, immune-checkpoint inhibitor; met., metastasis.
